# Evaluation of Mercury Contamination in Fungi *Boletus* Species from Latosols, Lateritic Red Earths, and Red and Yellow Earths in the Circum-Pacific Mercuriferous Belt of Southwestern China

**DOI:** 10.1371/journal.pone.0143608

**Published:** 2015-11-25

**Authors:** Jerzy Falandysz, Ji Zhang, Yuan-Zhong Wang, Martyna Saba, Grażyna Krasińska, Anna Wiejak, Tao Li

**Affiliations:** 1 Laboratory of Environmental Chemistry & ecotoxicology, Gdańsk University, Gdańsk, Poland; 2 Institute of Medicinal Plants, Yunnan Academy of Agricultural Sciences, Kunming, Yunnan, China; 3 Yunnan Technical Center for Quality of Chinese Materia Medical, Kunming, China; 4 Yuxi Normal University, Yuxi, Yunnan, China; University of Vigo, SPAIN

## Abstract

For the first time, highly elevated levels of mercury (Hg) have been documented for several species of the edible Fungi genus *Boletus* growing in latosols, lateritic red earths, and red and yellow earths from the Yunnan province of China. Analysis of Hg concentrations in the genus suggests that geogenic Hg is the dominant source of Hg in the fungi, whereas anthropogenic sources accumulate largely in the organic layer of the forest soil horizon. Among the 21 species studied from 32 locations across Yunnan and 2 places in Sichuan Province, the Hg was found at elevated level in all samples from Yunnan but not in the samples from Sichuan, which is located outside the mercuriferous belt. Particularly abundant in Hg were the caps of fruiting bodies of *Boletus aereus* (up to 13 mg kg^-1^ dry matter), *Boletus bicolor* (up to 5.5 mg kg^-1^ dry matter), *Boletus edulis* (up to 22 mg kg^-1^ dry matter), *Boletus luridus* (up to 11 mg kg^-1^ dry matter), *Boletus magnificus* (up to 13 mg kg^-1^ dry matter), *Boletus obscureumbrinus* (up to 9.4 mg kg^-1^ dry matter), *Boletus purpureus* (up to 16 mg kg^-1^ dry matter), *Boletus sinicus* (up to 6.8 mg kg^-1^ dry matter), *Boletus speciosus* (up to 4.9mg kg^-1^ dry matter), *Boletus tomentipes* (up to 13 mg kg^-1^ dry matter), and *Boletus umbriniporus* (up to 4.9 mg kg^-1^ dry matter). Soil samples of the 0–10 cm topsoil layer from the widely distributed locations had mercury levels ranging between 0.034 to 3.4 mg kg^-1^ dry matter. In Yunnan, both the soil parent rock and fruiting bodies of *Boletus* spp. were enriched in Hg, whereas the same species from Sichuan, located outside the mercuriferous belt, had low Hg concentrations, suggesting that the Hg in the Yunnan samples is mainly from geogenic sources rather than anthropogenic sources. However, the contribution of anthropogenically-derived Hg sequestered within soils of Yunnan has not been quantified, so more future research is required. Our results suggest that high rates of consumption of *Boletus* spp. from Yunnan can deliver relatively high doses of Hg to consumers, but that rates can differ widely because of large variability in mercury concentrations between species and locations.

## Introduction

Mercury is a ubiquitous trace element in the Earth’s crust. In some regions of the world, soils are enriched in Hg in the form of HgS, because of geochemical anomalies causing mercuriferous belts [1–3]. Today, the surface layer of forest and mountain topsoils worldwide is also usually enriched in Hg due to atmospheric deposition from anthropogenic sources [4–6]. This anthropogenically-caused enrichment of mercury in the organic layer of topsoils is a serious environmental concern, with potential negative impacts on both the environment and human health [7]. Mercury typically occurs in biota and foods in trace amounts both in the form of an inorganic (Hg^+/2+^) compounds and methylmercury, (MeHg, CH_3_Hg^+^), which is a persistent and highly toxic compound that is the most common organic form of Hg. The ongoing process of environmental spread of Hg because of anthropogenic activities is of consequence probably not only for the forest topsoil but also for biota, especially marine organisms and upper trophic-level species susceptible to bio-magnification [8].

Mercury is a semi-volatile metal and all its molecular forms are hazardous to human. The molecular forms that are such as HgSe (mineral tiemannite), HgS (mineral cinnabar), and little relevant environmentally Hg_2_Cl_2_ (calomel) are considered “safe” because they all have low solubility in water; however, following ingestion they dissociate and/or are more soluble in the highly acidic pH of gastric fluid after ingestion than in the water of a laboratory tube.

Although HgS is an ingredient in some medicinal preparations, including in Chinese Traditional Medicine [9], mice exposed to HgS experienced symptoms of neurotoxicity [10]. Nevertheless, little information exists on the possible Hg intake and risks associated with the consumption of mercury-contaminated medicine and foodstuff, such as edible mushrooms or herbs from the mercuriferous belts [11,12].

The ubiquity of Hg in the environment and its occurrence in food has resulted in low-level dietary intake of certain inorganic forms of Hg and MeHg, which are common trace-compounds in foods. At a regional scale, because of anthropogenic pollution (e.g. Minamata Bay) or geology (mercuriferous belts), exposure can be elevated for MeHg, as well as inorganic Hg, while beneficious Se in food chain could be in deficit [13–16]. Traditionally, daily meals often include wild-grown mushrooms as a small ingredient. Annual rates of intake of wild mushrooms are highly variable across different regions of the world, varying with cultural and family traditions in places such as the Czech Republic, Finland, the Yunnan of China, England, and Poland [17]. Seafood is viewed as a “source” of Hg to humans but is not a wild-grown and tasty mushroom, which among biota often is the best accumulator of Hg from soil. Hence, mushrooms could be an important local source of Hg to humans and the chemical form of Hg could be a crucial factor mediating the effects of mercury-contaminated mushrooms on human health.

For example, in fish muscles Hg occurs nearly totally in the form of MeHg bound to thiols (-SH) of cysteine (MeHg-cys) in proteins [16], and this is highly different when compared so far to data published for wild-grown mushrooms [8]. Fish is considered a major source of MeHg in humans but this can depend on the location and compositional structure of a diet (ingredients and their geographical origin). For example, for some people in the Guizhou Province of China neither, rice, rather than seafood, is the major source of dietary MeHg [18].

Edible wild grown mushrooms that grew in locations far from industrial and urban regions can be contaminated with Hg. This is because many mushrooms efficiently uptake this element from soil substratum underneath the fruiting bodies. For example, *Macrolepiota procera* (Parasol Mushroom) and *Boletus edulis* (King Bolete) are species whose mycelium efficiently absorbs Hg from the soil substratum and accumulate it in fruiting bodies—frequently at level > 3.0 mg kg^-1^ dm in areas with Hg in topsoil well below 0.05 mg kg^-1^ dm [19–36]. The Hg concentration of fruiting bodies of several popular edible mushrooms foraged from “pristine” European forests ranges from 0.27±0.07 to 8.4±7.4 mg kg^-1^ dry matter (with mean ranging from 0.027 to 0.84 mg kg^-1^ wet weight; assuming 90% moisture content) [37]. Genus *Boletus* has many species [38]. Some *Boletus* species are called “real boletes”, *such as*. *Boletus aereus*, *B*. *edulis*, *B*. *pinophilus*, and *B*. *reticulatus*, which have been found to be rich in Hg (and Se), while other species have been found to be much less in Hg (and Se) when compared to the “real boletes” (e.g. Bay Bolete *B*. *badius* (earlier called *Xerocomus badius*), Larch Bolete *Suillus grevillei*, Variegated Bolete *S*. *variegatus*) [35,39].

Mushrooms growing in places with elevated concentrations of Hg in the topsoil due to cinnabar (HgS) mining, processing of the non-ferrous metals, and other kinds of Hg hot spots usually contain elevated concentrations of Hg that is up to 10~100 fold greater than the amounts found in background areas (e.g., researchers found 20±42 mg Hg kg^-1^ dm in *Cantharellus cibarius*, 23±24 mg kg^-1^ dm in *M*. *procera*, and 52±61 mg kg^-1^ dm in *S*. *grevillei*) [40–49].

Although the primary source of many inorganic compounds for fungi seem to be from the substratrum (e.g. decaying litter, organic or mineral layer of soil, and dead or living vegetation), the movement of water and vertical migration of water soluble compounds through the soil horizon can also matter. Given the huge diversity of mushrooms (macrofungi), species-specific genetic features, in addition to the mycorrhizal/saprophytic lifestyle, likely influence the uptake and sequestration of certain mineral components in fruiting body of a given fungus. Some fungi also have rhizomorphs—root-like structures that enhance the uptake of water and water-soluble compounds [50]. For example, airborne pollutants such as Hg or radionuclide ^134/137^Cs are well accumulated by some species with shallow mycelia—Hg by *Gymnopus erythropus* and *Marasmius dryophilus*[4]. The nuclides ^134/137^Cs are better accumulated by *Cortinarius* spp., which richer in the stable caesium (^133^Cs) [51,52].

It is not well known which compound of Hg is the major constituent of total Hg in mushrooms and where it is located in the fruiting body and/or cells. The highly toxic MeHg is considered to be a minor fraction (< 5%) of the total Hg in mushrooms, although MeHg can be more efficiently accumulated in fruiting bodies than inorganic forms of Hg that dominate in flesh of mushrooms [8,47,53]. A low-level exposure to MeHg/total Hg is considered to not have substantial negative health consequences because of the protection provided by dietary selenium (Se) [54]. The beneficial role of Se, which can protect the cells from toxic action by Hg, has been explained based on the replacement of Se in the selenocysteine comprising selenoenzymes (e.g. gluthatione peroxidase; GPx) by the Hg in CH_3_Hg^+^ bound covalently to thiol (-SH) of cysteine (MeHg-cys) and the formation of a strong bond of Hg-Se in Se-Hg-cys complex [16,54]. The Se-Hg-cys complex is further degraded in lysosome to mercury-selenide (HgSe) and this can lead to weakening of an activity of selenoenzymes and a deficit in the body pool of Se, which is necessary for selenoenzyme synthesis.

Apart from Se there are also other possible ligands for Hg in fruiting bodies of mushrooms like thiols (-SH) in amino acids. Cysteine is typical but a minor component of the exogenous amino acids in mushrooms such as *Pleurotus ostreatus* and *Agaricus bisporus* [55]. Hence, cysteine is a possible ligand for MeHg in mushrooms. The seleno-compounds that have been found in mushrooms are selenocysteine, selenomethionine, Se-methylselenocysteine, and selenite, as well as several unidentified compounds. The concentrations of these compounds in mushrooms vary widely between species [56]. Sulphur [57], Se [58,59], and Hg [39,60] are the elements specifically abundant in some *Boletus* spp. Hence, it is possible that a bulk of Hg accumulated by mushrooms can be bound by S other than -SH of cysteine or by Se; however, evidence is lacking. A recent study showed that the majority of Hg contained in fruiting bodies does not leach during blanching, which suggesting it may strongly bind to functional groups or be present as compounds that do not easily dissolve [61].

The latosols, lateritic red earths, and red and yellow earths in southwest China showed geochemically-elevated concentrations of Hg when compared to some other regions in the country [62]. Nevertheless, there is a lack of studies and data on the influence of the mercuriferous belt on the accumulation and concentration of Hg in wild grown mushrooms, which are abundant and popular foods in Yunnan. This study aimed to get an insight into the accumulation, distribution and probable dietary intake of Hg contained in 21 species of *Boletus* mushrooms collected in 32 locations across Yunnan Province and 2 locations in Sichuan Province in the southwestern China. Topsoil samples underneath the fruiting bodies were collected from certain locations alongside the mushrooms. An attempt was also made to assess the exposure to Hg contained in those mushrooms using established safety criteria.

## Materials and Methods

### Mushrooms and topsoil samples

**Fig 1:**
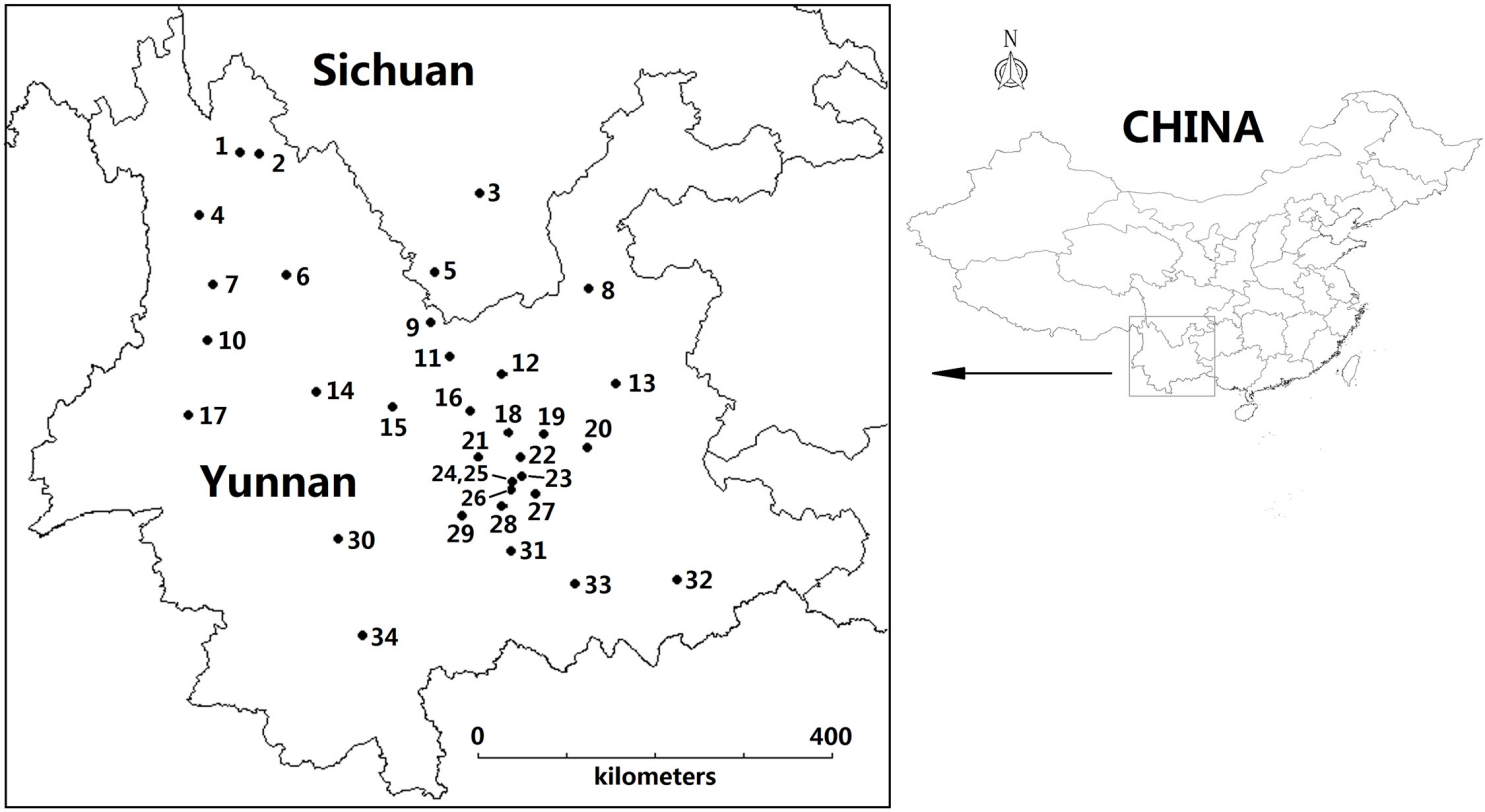
Localization of the sampling sites (1–34; for details see in Table 1). The figure was created by DIVA-GIS 7.5 software.

In order to investigate mushrooms representative of Yunnan, we chose species representing widely distributed locations and collected several individuals from a species in a given location, which were combined into composite samples for chemical analysis (Fig 1). No specific permits were required for the described field studies. No endangered or protected species were sampled, and the localities where the samples came from are not protected in any way. Matured mushrooms with small fruiting bodies are usually pooled before determination of trace element concentrations [45,46,63]. Examination of such composite samples allows for a substantial reduction of costs with limited loss of information and prevention of a bulk of sample material for other analyses [64]. Hence, it is possible to obtain representative information on the concentration of a given chemical element in fruiting bodies based on a composite sample instead of the examination of individual specimens (15 individual samples is the minimum required per location/population) [65].

In total, 968 specimens of 21 species of edible mushrooms of genus *Boletus* were collected in forests from 32 locations across Yunnan and 2 places in Sichuan Province of China during the collection season (June-September) in 2011–2014 (Fig 1). Soil samples of the forest topsoil layer (0–10 cm) beneath the fruiting bodies were also collected. The species collected were *Boletus aereus* Fr. ex Bull, *Boletus amygdalinus* (Thiers) Thiers, *Boletus auripes* Peck, *Boletus bicolor* Peck, *Boletus brunneissimus* Chiu, *Boletus calopus* Fr, *Boletus edulis* Bull., *Boletus ferrugineus* Schaeff, *Boletus fulvus* Peck, *Boletus griseus* Frost., *Boletus impolitus* Fr., *Boletus luridiformis* Rostk., *Boletus luridus* Schaoff.:Fr., *Boletus magnificus* Chiu., *Boletus obscureumbrinus* Hongo, *Boletus pallidus* Frost, *Boletus purpureus* Fr., *Boletus sinicus* W.F.Chiu, *Boletus speciosus* Forst., *Boletus tomentipes* Earle and *Boletus umbriniporus* Hongo [38]. The collected fruit bodies were in good “edible” body condition (not injured by insects) and well developed (old and “baby” specimens were not selected). Any visible plant vegetation and soil debris were cleaned off of the fresh fruit bodies using a plastic knife. To get insight into the distribution of Hg between two major morphological parts of the fruit bodies, each specimen was separated into cap (with skin) and stipe. Next, the individual cap and stipe samples were sliced using a plastic knife and pooled accordingly to obtain representative composite samples representing each species (with 5 to 21 individuals per pool), sampling place and time of collection (Table 1).

**Table 1:** Summary of results of Hg determination in fungal certified reference materials (mg kg^-1^ dry matter).

Certified reference material symbol	Declared Hg concentration	Determined Hg concentration
CS-M-1	0.174±0.018	0.18 ± 0.01 (n = *13*)
CS-M-2	0.164±0.004	0.16 ± 0.01 (n = *8*)
CS-M-3	2.849±0.104	2.8±0.0 (n = *5*)
CS-M-4	0.465±0.024	0.45±0.03 (n = *14*)

Thereafter, the mushroom samples were placed into the plastic basket of the electrically heated commercial dryer for vegetables and dried at 65°C to constant mass. Dried fungal materials were pulverized in a porcelain mortar and kept in brand new sealed polyethylene bags under dry conditions. The soil samples, free of any visible organisms, small stones, sticks and leaves were air dried at room temperature for several days under clean conditions and further dried at 65°C to constant mass. Next, the soil samples were ground in a porcelain mortar, sieved through a pore size of 2 mm plastic sieve and kept similarly to fungal materials.

### Mercury determinations

All the reagents used in this study were of analytical reagent grade, unless otherwise stated. Double distilled water was used for the preparation of the solutions. Mercury standard solution of 1.0 mg Hg mL^-1^ was obtained from the 10 mg mL^-1^ standard stock solution. Blank and 100, 150, and 200 μL of 1.0 mg mL^-1^ Hg standard solutions were injected into the analyzer for the construction of a calibration curve, which was prepared each week.

The determinations of total Hg concentration of fungal and soils samples was performed using cold-vapour atomic absorption spectroscopy (CV-AAS) by a direct sample thermal decomposition coupled with gold wool trap of Hg and its further desorption and quantitative measurement at wavelength of 296 nm. Each sample was examined at least in duplicate and most of the samples, because of unexpectedly high Hg concentrations, were examined in triplicate. The analytical instrument used was a mercury analyzer (MA-2000, Nippon Instruments Corporation, Takatsuki, Japan) equipped with an auto-sampler, and operated in low or high modes, as appropriate [66,67].

A running analytical control and assurance quality (AC/AQ) was performed through the analysis of blank samples and certified reference materials such as CS-M-1 (dried mushroom powder *Suillus bovinus*), CS-M-2 (dried mushroom powder *Agaricus campestris*), CS-M-3 (dried mushroom powder *Boletus edulis*), and CS-M-4 (dried mushroom powder *Leccinum scabrum*) produced by the Institute of Nuclear Chemistry and Technology, Warsaw, Poland (Table 1).

The limit of detection (LOD) of this study was 0.003 mg Hg/kg dm, and the quantification limit (LOQ) was 0.005 mg Hg kg^-1^ dm. One blank sample and one certified reference material sample were examined with each set of 3–5 samples studied.

The bioconcentration factor (BCF) value (calculated for mushrooms as the concentration quotient of fruiting body/cap or stipe to that of the underlying soil substrate) is used to estimate possible influence. BCF allows us to estimate the elements that are actively accumulated (BCF > 1) and those not actively accumulated, i.e. excluded (BCF < 1).

## Results and Discussion

### Hg in fruiting bodies

There is a scarcity of data on the accumulation and distribution of Hg in wild grown fungi collected from the soils of the circum-Pacific mercuriferous belt of Yunnan [11,12] or outside of the belt in China [4,68,69]. In general, the concentration of Hg in caps and stipes differed substantially both between Yunnan locations and between species (Fig 1, Table 2). The overall range of Hg concentrations in composite samples of the caps of *Boletus* spp. in this study was from 0.13 mg kg^-1^ dm up to 22 mg kg^-1^ dm, the record high value reported for any mushroom collected from the area (an area previously considered as background due to receiving limited Hg pollution). In the stipes, the values of Hg ranged from 0.12 mg kg^-1^ dm to 8.4 mg kg^-1^ dm (Table 2).

**Table 2:** Mercury concentration in mushrooms of genus *Boletus* and soil substratum from the China and Poland, quotient of Hg concentration in caps to stipes (Q_C/S_), and quotient of Hg concentration in cap/stipe to soil beneath the fruiting bodies (BCF; bioconcentration factor).

Species, location* and year of collection	*n*	Hg (mg kg^-1^ dm)			Q_C/S_	BCF	
		Fruiting bodies		Soil		Cap	Stipe
		Cap	Stipe				
***Boletus amygdalinus* (Thiers) Thiers**							
[32]*Dongshan, Wenshan; 2012	(10)**	0.63	0.30	0.22	2.1	2.9	1.4
***Boletus aereus* Fr. ex Bull**							
[9] Yongren, Chuxiong; 2012	(10)	13	4.2	0.68	3.1	19	6.2
[32] Dongshan, Wenshan; 2012	(7)	1.6	0.96	0.22	1.7	7.3	4.4
***Boletus auripes* Peck**							
[21] Yimen, Yuxi; 2011	(9)	1.6	0.95	WD	1.7	WD	WD
[12] Wuding, Chuxiong; 2011	(11)	1.8	1.1	WD	1.6	WD	WD
***Boletus bicolor* Peck**							
[27] Jiangchuan, Yuxi; 2012	(10)	0.89	0.66	0.19	1.4	4.7	3.5
[26] Dayingjie, Yuxi; 2014	(7)	5.5	2.5	WD	2.2	WD	WD
***Boletus brunneissimus* Chiu**							
[18] Anning, Kunming; 2012	(9)	1.2	0.67	0.29	1.8	4.1	2.3
***Boletus calopus* Fr**							
[13] Malong, Qujing; 2013	(10)	0.86	0.38	0.073	2.3	12	5.2
***Boletus edulis* Bull: Fr**							
[22] Jinning, Kunming; 2011	(11)	2.1	1.3	WD	1.6	WD	WD
[18] Anning, Kunming; 2012	(10)	4.8	1.8	WD	2.7	WD	WD
[20] Shilin, Kunming; 2012	(10)	13	6.9	1.1	1.9	12	6.3
[21] Yimen, Yuxi; 2011	(12)	4.5	1.4	WD	3.2	WD	WD
[21] Yimen, Yuxi; 2012	(10)	6.3	2.4	0.91	2.6	6.9	2.6
[21] Yimen, Yuxi; 2012	(10)	4.5	2.2	0.80	2.0	5.6	2.8
[26] Dayingjie, Yuxi; 2013	(14)	22	8.4	3.4	2.6	6.5	2.5
[29] Xinping, Yuxi; 2014	(15)	3.9	1.3	0.35	3.0	11	3.7
[24] Jiulongchi, Yuxi; 2014	(21)	3.6	1.3	0.15	2.8	24	8.7
[15] Nanhua, Chuxiong; 2011	(7)	7.3	3.9	WD	1.9	WD	WD
[15] Nanhua, Chuxiong; 2011	(10)	5.3	2.4	WD	2.2	WD	WD
[15] Nanhua, Chuxiong; 2013	(10)	3.0	1.1	0.27	2.7	11	4.1
[15] Nanhua, Chuxiong; 2013	(13)	3.5	1.5	0.12	2.3	29	12
[2] Pudacuo, Diqing; 2012	(10)	1.6	0.96	0.20	1.7	8.0	4.8
[14] Midu, Dali; 2012	(10)	2.7	0.85	0.35	3.2	7.7	2.4
[6] Heqing, Dali; 2012	(7)	4.4	1.9	WD	2.3	WD	WD
[4] Weixi, Diqing; 2012	(7)	17	6.4	1.3	2.7	13	4.9
[17] Longyang region, Baoshan; 2012	(10)	15	8.2	2.1	1.8	7.1	3.9
[32] Dongshan, Wenshan; 2012	(7)	11	4.5	1.2	2.4	9.2	3.8
[30] Zhenyuan, Puer; 2014	(5)	3.5	1.4	0.13	2.5	27	11
***Boletus ferrugineus* Schaeff**							
[2] Pudacuo, Diqing; 2012	(10)	0.92	0.46	0.21	2.0	4.4	2.2
[7] Lanping, Nujiang; 2012	(7)	0.72	0.54	WD	1.3	WD	WD
[12] Wuding, Chuxiong; 2011	(10)	3.1	1.7	WD	1.8	WD	WD
[19] Kunming city; 2011	(8)	0.66	0.53	WD	1.2	WD	WD
[21] Yimen, Yuxi; 2012	(9)	1.3	0.76	WD	1.7	WD	WD
[26] Dayingjie, Yuxi; 2014	(15)	7.7	7.2	WD	1.1	WD	WD
[20] Shilin, Kunming; 2012	(9)	0.27	0.22	WD	1.2	WD	WD
***Boletus fulvus* Peck**							
[14] Midu, Dali; 2012	(10)	1.6	0.72	0.70	2.2	2.3	1.0
***Boletus griseus* Frost.**							
[19] Kunming city; 2011	(8)	0.66	0.38	WD	1.7	WD	WD
[18] Anning, Kunming; 2012	(7)	4.9	0.81	WD	6.0	WD	WD
[20] Shilin, Kunming; 2012	(10)	1.3	0.60	0.22	2.2	5.9	2.7
[17] Longyang region, Baoshan; 2012	(10)	3.0	2.9	0.83	1.0	3.6	3.5
[27] Jiangchuan, Yuxi; 2012	(10)	1.4	0.82	0.15	1.7	9.3	5.5
[26] Dayingjie, Yuxi; 2014	(13)	1.9	0.59	WD	3.2	WD	WD
[14] Midu, Dali; 2012	(10)	1.2	0.62	0.13	1.9	9.2	4.8
[13] Malong, Qujing; 2012	(7)	3.4	0.87	WD	3.5	WD	WD
[16] Lufeng, Chuxiong; 2013	(10)	1.6	1.2	0.10	1.3	16	12
[16] Lufeng, Chuxiong; 2013	(17)	2.0	0.72	0.083	2.8	24	8.7
[30] Zhenyuan, Puer; 2014	(6)	2.2	2.2	WD	1.0	WD	WD
***Boletus impolitus* Fr.**							
[19] Kunming city; 2011	(10)	1.9	0.89	WD	2.1	WD	WD
[21] Yimen, Yuxi; 2012	(8)	1.9	1.0	0.43	1.9	4.4	2.3
***Boletus luridiformis* Rostk.**							
[13] Malong, Qujing; 2013	(10)	2.8	1.1	0.21	2.5	13	5.2
***Boletus luridus* Schaoff.:Fr.**							
[20] Shilin, Kunming; 2012	(8)	2.1	0.67	WD	3.1	WD	WD
[14] Midu, Dali; 2012	(8)	3.0	1.8	0.32	1.7	9.4	5.6
[11] Yuanmou, Chuxiong; 2012	(8)	11	4.2	1.2	2.6	9.2	3.5
***Boletus magnificus* Chiu.**							
[27] Jiangchuan, Yuxi; 2012	(10)	1.3	0.67	0.13	1.9	10	5.2
[21] Yimen, Yuxi; 2012	(10)	2.7	1.2	WD	2.2	WD	WD
[21] Yimen, Yuxi; 2012	(7)	1.3	0.87	0.29	1.5	4.5	3.0
[26] Dayingjie, Yuxi; 2014	(8)	13	5.1	WD	2.5	WD	WD
[14] Midu, Dali; 2012	(9)	2.4	0.84	0.32	2.9	7.5	2.6
***Boletus obscureumbrinus* Hongo**							
[34] Simao region, Puer; 2013	(10)	6.3	3.6	0.53	1.8	12	6.8
[34]Simao region, Puer; 2013	(21)	9.4	6.0	0.43	1.6	22	14
***Boletus pallidus* Frost**							
[18] Anning, Kunming; 2012	(10)	1.3	0.84	0.19	1.6	6.8	4.4
***Boletus purpureus* Fr.**							
[21] Yimen, Yuxi; 2012	(10)	3.0	1.4	WD	2.1	WD	WD
[21] Yimen, Yuxi; 2012	(8)	2.1	1.4	WD	1.5	WD	WD
[10] Yunlong, Dali; 2012	(10)	7.4	5.6	2.6	1.3	2.9	2.2
[7] Lanping, Nujiang; 2012	(12)	16	6.1	2.4	2.6	6.7	2.5
***Boletus sinicus* W.F. Chiu**							
[13] Malong, Qujing; 2013	(10)	2.4	0.93	0.16	2.6	15	5.8
[24] Jiulongchi, Yuxi; 2013	(8)	6.8	4.1	0.22	1.7	31	17
***Boletus speciosus* Forst.**							
[18] Anning, Kunming; 2012	(10)	2.7	1.1	WD	2.5	WD	WD
[18] Anning, Kunming; 2012	(10)	1.8	0.79	0.52	2.3	3.5	1.5
[21] Yimen, Yuxi; 2012	(10)	0.90	0.78	WD	1.2	WD	WD
[28] Eshan, Yuxi; 2013	(6)	2.1	1.0	WD	2.1	WD	WD
[28]Eshan, Yuxi; 2014	(12)	4.8	1.5	0.87	3.2	5.5	1.7
[25] Beicheng, Yuxi, 2014	(12)	0.85	1.0	0.096	0.85	8.8	10
[26] Dayingjie, Yuxi; 2013	(19)	2.7	1.3	0.20	2.1	8.7	6.5
[17] Longyang region, Baoshan; 2012	(10)	2.7	1.5	0.31	1.8	8.7	4.8
[11] Yuanmou, Chuxiong; 2012	(10)	4.9	1.2	0.40	4.1	12	3.0
[8] Huize, Qujing; 2013	(10)	3.9	1.7	WD	2.3	WD	WD
[8] Huize, Qujing; 2014	(6)	2.1	1.0	WD	2.1	WD	WD
***Boletus tomentipes* Earle**							
[28] Eshan, Yuxi; 2011	(10)	12	3.5	WD	3.4	WD	WD
[28] Eshan, Yuxi; 2011	(9)	5.1	4.3	WD	1.2	WD	WD
[28] Eshan, Yuxi; 2011	(7)	3.5	1.8	0.24	1.9	15	7.5
[28] Eshan, Yuxi; 2014	(12)	3.9	3.1	0.31	1.3	13	10
[26] Dayingjie, Yuxi; 2014	(14)	13	6.2	0.46	2.1	28	14
[21] Yimen, Yuxi; 2011	(9)	1.7	2.9	0.21	0.59	8.1	14
[29] Xinping, Yuxi; 2014	(13)	9.2	6.6	0.13	1.4	71	51
[34] Simao region, Puer; 2011	(10)	0.58	1.2	WD	0.48	WD	WD
[15] Nanhua, Chuxiong; 2011	(8)	4.5	3.3	WD	1.4	WD	WD
[33] Gejiu, Honghe; 2012	(7)	3.7	2.3	0.42	1.6	8.8	5.5
[1] Shangri-la, Diqing; 2011	(9)	0.13	0.12	WD	1.1	WD	WD
[31] Shiping, Honghe; 2012	(8)	5.4	2.8	0.98	1.9	5.5	2.9
[3] Dechang, Sichuan; 2012	(9)	0.19	0.12	0.034	1.6	5.6	3.5
[5] Panzhihua, Sichuan; 2012	(10)	0.29	0.17	0.055	1.7	5.3	3.1
[6] Heqing, Dali; 2012	(7)	0.45	0.60	0.14	0.75	3.2	4.3
***Boletus umbriniporus* Hongo**							
[21] Yimen, Yuxi; 2011	(10)	0.64	0.46	WD	1.4	WD	WD
[21] Yimen, Yuxi; 2012	(7)	1.4	0.59	0.43	2.4	3.3	1.4
[23] Huangcaoba, Yuxi; 2011	(8)	1.3	0.86	0.64	1.5	2.0	1.3
[26] Dayingjie, Yuxi; 2013	(8)	1.8	0.79	WD	2.3	WD	WD
[17] Longyang region, Baoshan; 2012	(7)	3.8	1.4	0.46	2.7	8.3	3.0
[14] Midu, Dali; 2012	(8)	0.54	0.94	0.41	0.57	1.3	2.3
[31] Shiping, Honghe; 2012	(9)	0.73	0.48	0.35	1.5	2.1	1.4
[11] Yuanmou, Chuxiong; 2011	(10)	4.9	2.2	0.25	2.2	20	8.8
[8] Huize, Qujing; 2013	(10)	1.6	0.99	WD	1.6	WD	WD
[8] Huize, Qujing; 2013	(8)	1.8	0.79	WD	2.3	WD	WD
[15] Nanhua, Chuxiong; 2013	(10)	1.7	0.86	0.20	2.0	8.5	4.3
[15] Nanhua, Chuxiong; 2013	(9)	1.0	0.49	WD	2.0	WD	WD

Notes:

*(see Fig 1);

**(number of individuals);

WD (without data)

There is a high scarcity of data on the occurrence of Hg in *Boletus* mushrooms foraged in Yunnan. Apart from a highly contaminated *B*. *edulis* specimen from the location Dayingie in the central region of Yunnan (22 mg kg^-1^ dm in caps), mushrooms with high Hg concentrations were found in: *B*. *aereus* from Yongren in the Chuxiong Autonomous Prefecture (13 mg Hg kg^-1^ dm), *B*. *bicolor* from the Dayingie in the Yuxi City (at 5.5 mg kg^-1^ dm), *B*. *ferrugineus* from the Dayingie in the Yuxi City (at 7.7 mg kg^-1^ dm), *B*. *luridus* from the Yuanmou in the Chuxiong Autonomous Prefecture (at 11 mg kg^-1^ dm), *B*. *magnificus* from the Dayinjie in the Yuxi City (at 13 mg kg^-1^ dm), *B*.*obscureumbrinus* from the Simao region in the Pu'er City (at 9.4 mg kg^-1^ dm), *B*. *purpureus* from the Lanping in the Nujiang Autonomous Prefecture (at 16 mg kg^-1^ dm), *B*. *sinicus* from the Jiulongchi in the Yuxi City (at 6.8 mg kg^-1^ dm), *B*. *speciosus* from the Yuanmou in the Chuxiong Autonomous Prefecture (at 4.9 mg kg^-1^ dm), *B*. *tomentipes* from the Dayingjie in the Yuxi City (at 13 mg kg^-1^ dm), and *B*.*umbriniporus* from the Yuanmou in the Chuxiong Autonomous Prefecture (at 4.9 mg kg^-1^ dm) (Table 2).

Because of the large number of *Boletus* species examined here, for clarity of presentation the results that follow are alphabetically ordered by species names.

#### B. aereus

*B*. *aereus* prefers a warm climate. This fungus showed a capacity for accumulation of Hg in the fruiting bodies and in this study the maximum was 13 mg Hg kg^-1^ dm in caps and 4.2 mg kg^-1^ dm in stipes from the Yongren in the Chuxiong Autonomous Prefecture in the northern part of Yunnan. For the specimens from the south of Yunnan in Dongshan in the Wenshan Autonomous Prefecture, the values of Hg were an order of magnitude lower (Table 1).

Our results well agree with data on Hg in *B*. *aereus* from other parts of the world, which are few and relate to individuals collected from the southern regions of Europe. Namely, in a recent study *B*. *aereus* from Seville Spain showed Hg at 10 ± 3 mg kg^-1^ dm in the caps and 8 ± 3 mg kg^-1^ dm in the stipes [70]. Another study from Lugo, Galicia Spain showed the Hg at 4.6 ± 2.3 mg kg^-1^ dm in hymenophore and at 3.3 ± 1.5 mg kg^-1^ dm in the rest of the fruiting bodies of *B*. *aereus* [34], while the mean value of Hg in whole fruiting bodies from the Reggio de Emilia localization in Italy was 3.29 mg kg dm^-1^ [71].

The topsoil samples beneath the fruiting bodies of *B*. *aereus* contained Hg at 0.68 mg kg^-1^ dm in the Yongren location and 0.22 mg kg^-1^ dm in the Dongshan location, while the BCF values of Hg were high for both areas, *i*.*e*. BCF at 19 and 7.3 for caps and at 6.2 and 4.4 for stipes (although our sample size was only two per material). These high values of BCF demonstrate a high capacity of the species to bio-include Hg even if the element concentration in soil was elevated. The value of BCF showed that Hg up-take and sequestration by *B*. *aereus*is is higher for soils with greater concentrations of Hg in the mineral horizon (Table 1).

Melgar et al. [35] reported a very high potential of *B*. *aereus* to bioconcentrate Hg (BCF at 315–424) for low polluted soil substratum—the Hg concentration of soil in Lugo was at ~ 0.01 mg kg^-1^ dm. It is worth noting that there are regions in both Italy and Spain that are under the impact of mercuriferous belts [1]. Nevertheless, the Hg in soil substratum of *B*. *aereus* at the Lugo locale was ~20 to 60-fold less than the soils in Yunnan. Unfortunately, there is no information on Hg in soils substratum for the Reggio de Emilia or Sevilla.

#### *B*. *amygdalinus*, *B*. *auripes*, *B*. *bicolor*, *B*. *brunneissimus*, and *B*. *calopus*

Samples of *B*. *amygdalinus* (former name *Xerocomus amygdalinus*), *B*. *auripes*, *B*. *bicolor*, *B*. *brunneissimus*, and *B*. *calopus*, were available from a few places only. The pooled samples showed a range of Hg concentrations in the caps, with 0.63 mg kg^-1^ dm for *B*. *amygdalinus* up to 5.5 mg kg^-1^ dm for *B*. *bicolor*, and stipes were 50% less contaminated (Table 1). The relative high concentration of Hg in *B*. *bicolor* sampled from the Dayingjie region in the county of Yuxi can be attributed to the elevated concentration of the element in topsoil, as was noted for some other species from this region (Table 1). All of these five boletus species are common in Yunnan, but there is no information on the occurrence of Hg in fruiting bodies of these species from locations outside Yunnan.

*B*. *bicolor* sampled in the Sichuan Province of China from a place with Hg in topsoil of < 0.1 mg kg^-1^ dm contained 0.19 mg Hg kg^-1^ dm in caps [72]. No other information is available on accumulation and occurrence of Hg in *B*. *amygdalinus*, *B*. *auripes*, *B*. *bicolor*, *B*. *brunneissimus*, and *B*. *calopus*.

#### B. edulis

*B*. *edulis* from all places sampled in Yunnan showed “elevated” concentration of Hg, and the values ranged from 1.6 mg kg^-1^ dm to 22 mg kg^-1^ dm for the caps and from 0.85 mg kg^-1^dm to 8.2 mg kg^-1^ dm for the stipes. The median value of Hg for consignments of *B*. *edulis* for 20 places scattered across Yunnan was 4.5 mg kg^-1^ dm for caps and 1.9 mg kg^-1^ dm for stipes.

A few earlier data are also known on concentration of Hg, As, Cd, Pb, and Zn in *B*. *edulis* from Liangshan in Sichuan with Hg at 0.28 mg kg^-1^ dm and at 1.8 mg kg^-1^ dm in sample from mountains in Sichuan of China [68,69,73]. In another study in Sichuan, the Hg in caps of *B*. *edulis* was 0.38 and in stipes 0.16 mg kg^-1^ dm, while in soil substratum was < 0.1 mg kg^-1^ dm [72].

The *B*. *edulis* (King Bolete) from Europe has been well studied for Hg, many other trace elements, and macro elements [20–27,29–31,35,60,71,74–77]. From the studies mentioned it is known that in Europe *B*. *edulis* is efficient in the up-take and sequestration in flesh of Hg. For example, *B*. *edulis* collected from many background locations contained Hg ranging from 1.1±1.4 mg kg^-1^ dm to 7.6±3.1 mg kg^-1^ dm in the caps and from 0.82±0.71 to 3.8±1.8 mg kg^-1^ dm in the stipes in Poland; 7.9±0.3 mg kg^-1^ dm in a whole fruiting bodies from the Precambrian shale’s location in Bohemia of the Czech Republic and 2.7 (1.0–4.3) mg kg^-1^ dm in a whole fruiting bodies in the Reggio Emilia and from 1.9±1.0 to 4.5±1.0 mg kg^-1^ dm in Tuscany of Italy. This species collected in the region of Lugo in Galicia of Spain showed Hg in hymenophore of 3.3±2.4 mg kg^-1^ dm and in the rest of fruiting bodies of 2.0±1.2 mg kg^-1^ dm.

#### *B*. *fulvus*, *B*. *griseus*, *B*. *impolitus*, and *B*. *luridiformis*

These four species (*B*. *fulvus*, *B*. *griseus*, *B*. *impolitus*, and *B*. *luridiformus*) from Yunnan have not been so far studied for the accumulation and contamination with Hg and they are without the BCF values. They all can be considered as rich in Hg with maximum of up to 4.9 mg kg^-1^ dm in a pool of the caps of *B*. *griseus*, while they seem not so efficient in up-take and sequestration of Hg as *B*. *aereus* and *B*. *edulis*. In fruiting bodies of *B*. *griseus* sampled in 2006 from the Xichang region of the Sichuan Province, the Hg was usually much lower than determined in present study, *i*.*e*. at 0.34 and 0.94 mg kg^-1^ dm [69] or in caps at 0.10 mg kg^-1^ dm [72]. No other data on occurrence of Hg in *B*. *fulvus*, *B*. *griseus*, and *B*. *luridiformis* could be found in scientific literature. A specimen of *B*. *impolitus* sampled from the Pb-Zn mine area in the Lanping County in north-western region of Yunnan contained Hg in a whole fruiting body at 6.5 mg kg^-1^ dm [78].

Concentration of Hg determined in *B*. *impolitus* from Yunnan (Table 1) is almost the same value as reported recent in a study in Poland. For a collection of the fruiting bodies of *B*. *impolitus* from the localization situated far away from the mercuriferous belts in the north-eastern region of Poland where Hg in soil substratum was at 0.042±0.014 mg kg^-1^ dm, the concentration of Hg in the caps was at 1.8±0.6 mg kg^-1^ dm and in the stipes at 0.70±0.21 mg kg^-1^ dm [39]. The values of BCF calculated for caps and stipes of the individuals from Yunnan were respectively 4.4 and 2.3 (Table 1) and for individuals from the northern region of Poland were an order of magnitude higher, *i*.*e*. 47 and 17 (median) [39]. This implies on a much better absorption and sequestration by *B*. *impolitus* of Hg which contaminate the environment because of a global anthropogenic fallout of Hg (a major or sole source of Hg in topsoil/soil in Poland) than of the geogenic Hg from a geochemical anomaly.

#### *B*. *luridus*, *B*. *magnificus*, and *B*. *obscureumbrinus*

There are no previous records available on Hg in *B*. *luridus*, *B*. *magnificus*, and *B*. *obscureumbrinus* from China. In this study, the mushrooms *B*. *luridus* with up to 11 mg Hg kg^-1^ dm and *B*. *magnificus* with up to 13 mg Hg kg^-1^ are among those *Boletus* spp., which in several locations in Yunnan are highly contaminated with Hg. Two collections of individual samples of *B*. *obscureumbrinus* were available only from the Simao region in the Pu’er City on the south of the Yunnan and both mushrooms from consignments were rich in Hg, which ranged from 6.3 to 9.4 mg kg^-1^ dm in the caps and from 3.6 to 6.0 mg kg^-1^ dm in the stipes (Table 1).

In case of *B*. *luridus*, *B*. *magnificus*, and *B*. *obscureumbrinus* mushrooms, the only previous record available is for *B*. *luridus* collected from three spatially distantly distributed places in Europe, which showed Hg in the caps of 0.40±0.10 to 0.89±0.40 mg kg^-1^ dm and 0.15±0.06 to 0.39±0.16 mg kg^-1^ dm in stipes [39]. The Yunnan’s *B*. *luridus* mushroom with Hg in the caps of 2.1–11 and in the stipes of 0.67–4.2 mg kg^-1^ dm seems to pick-up Hg largely from the mineral layer of soil horizon that is around 10-100-fold more enriched in Hg (Table 1), when compared to concentration in topsoil substratum to *B*. *luridus* from Poland [39].

#### *B*. *pallidus*, *B*. *purpureus*, and *B*. *sinicus*

Mushroom *B*. *pallidus* was available from one place and showed Hg in the caps of 1.3 mg kg^-1^ dm. It can be classified into the group of the *Boletus* mushrooms in Yunnan, which are less contaminated with Hg—may be because of low bioconcentration potential for Hg compounds or because of low concentration of element in soils (< 0.20 mg kg^-1^ dm) at the sampling locations. Both *B*. *purpureus* and *B*. *sinicus* from the soils with elevated concentration of Hg, which showed contained from 2.4 to 4.3 mg Hg kg^-1^ dm, were substantially contaminated and concentration were up to 16 mg Hg kg^-1^ dm in the caps and up to 6.8 mg Hg kg^-1^ dm in the stipes (Table 1). No data are available from the scientific literature on Hg compounds in the fruiting bodies accumulation and distribution by *B*. *pallidus*, *B*. *purpureus* and, *B*. *sinicus*.

#### *B*. *speciosus*, *B*. *tomentipes*, and *B*. *umbriniporus*

The species such as *B*. *speciosus*, *B*. *tomentipes*, and *B*. *umbriniporus* are among the most popular edible fungi of genus *Boletus* in Yunnan. They contained Hg in fruiting bodies at a wide range of concentrations which was dependent on the geographical location. The *B*. *speciosus* with Hg in the caps ranged from 0.90 to 4.9 mg kg^-1^ dm, the *B*. *tomentipes* with from 0.13 to 13 mg kg^-1^ dm, and the *B*. *umbriniporus* with from 0.54 to 4.9 mg kg^-1^ dm can be classified among the species in this study that are substantially contaminated in view of the food toxicology philosophy.

In previous reports from the Sichuan Province of China, the reported concentration of Hg in whole fruiting bodies of *B*. *umbriniporus* from the Liangshan place was 0.18 mg kg^-1^ dm [73], and in caps and stipes of *B*. *umbriniporus* sampled elsewhere in the Sichuan, the Hg were 0.16 and 0.11 mg kg^-1^ dm for caps and stipes respectively [72].

### Bioconcentration factors

The soil samples from the widely distributed locations in Yunnan showed Hg in 0–10 cm layer at 0.073 to 3.4 mg kg^-1^ dm and most of the samples were well above 0.2 mg kg^-1^ dm, while in two places in Sichuan were at 0.034 to 0.055 mg kg^-1^ dm (Table 1). The overriding source of elevated concentration of Hg in the mineral layer of latosols, lateritic red earths and red and yellow earths of Yunnan in this study is geochemical anomaly due to occurrence of the circum-Pacific mercuriferous belt, while Hg deposited from the atmosphere is retained in a top 0–3 cm layer of forest soil of Yunnan, which is black and rich in decaying litter and humus with organic substances.

The red earths are the dominant highly weathered soil type in China and widely distributed in the hilly and mountainous regions of northern part of Yunnan, while the lateritic red earths are mainly distributed in areas bordering between the tropical and subtropical regions of southeast part of Yunnan [79]. There is scarcity of data on Hg concentration of soils in Yunnan. Wen and Chi [62] reported an average Hg concentration of 0.14 mg kg^-1^ dm (2,995 samples) in different types of sediments in southwest China (central and east Yunnan included), at 0.046 mg kg^-1^ dm (1,190 samples) from rain forest (south Yunnan included) and at 0.045 mg kg^-1^ dm (1,183 samples) from alpine valleys (northwest Yunnan included). In south and southwest China, median Hg concentrations in the C horizon and A horizons of soils (totally ~ 12,000 samples) are 0.076 mg kg^-1^ dm (C) and 0.086 mg kg^-1^ dm (A) in yellow earth, 0.044 mg kg^-1^ dm (C) and 0.069 mg kg^-1^ dm (A) in red earth, and 0.035 mg kg^-1^ dm (C) and 0.044 mg kg^-1^ dm (A) in latosolic red earth [62].

In some other studies in China, in the Sichuan Province, which is largely the farmland region, soils showed Hg in tea garden soil at 0.039 mg kg^-1^ dm (up to 0.37 mg kg^-1^ dm) [80], and at 0.15 mg kg^-1^ dm (0.003–0.71 mg kg^-1^ dm) in 239 samples of topsoil from the central region of the province [81]. There is no data available on speciation of Hg in soils of Yunnan. In the active Xunyang Hg mine area in the south of the Shaanxi Province in China, which is in northeast direction to Yunnan, the Hg and MeHg concentrations in riparian soils ranged respectively from 5.4 to 120 mg kg^-1^ dm and from 0.0012 to 0.011 mg kg^-1^ dm [3].

Mercury can occur in organic and mineral layer of topsoil in various forms [82], such as chelated (bound to organic substances), precipitated (in sulphide, carbonate, hydroxide, phosphate and others), specifically and non-specifically adsorbed (because of covalent and coordinative or electrostatic binding), and dissolved (free ion or soluble complex). In a study by Rodrigues et al. [83], the geogenic Hg in soils from a mining area (Hg at 0.92 to 37 mg kg^-1^ dm) was in a greater proportion as non-mobile forms that was less extractable, while more mobile and semi-mobile Hg were in urban and industrial soil, which were better extractable by 0.1M HCl or surrogates of human gastric fluid (pH 1.5) and human lung fluid (pH 7.4).

**Fig 2:**
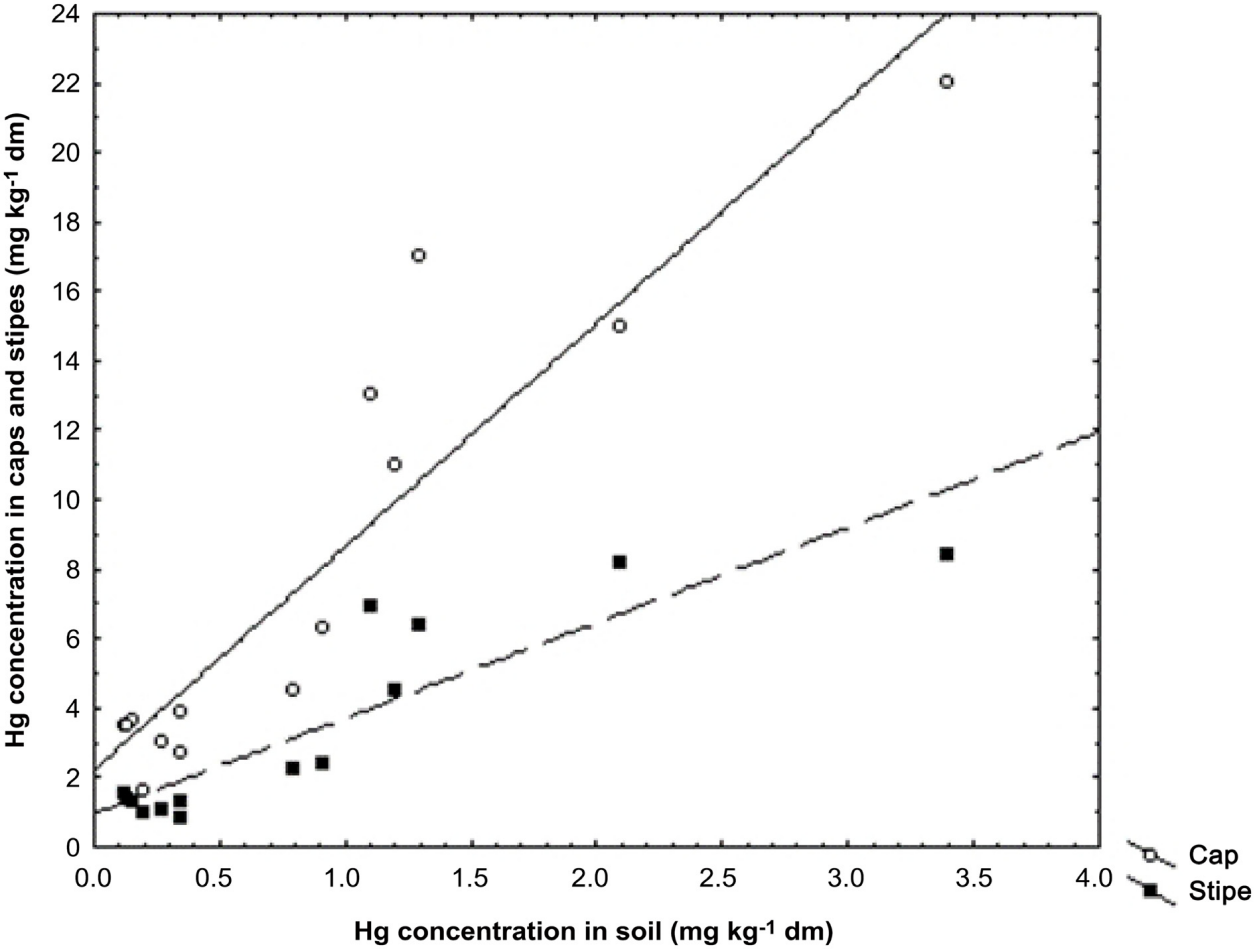
Relationships between Hg concentration in the caps (y = 2.2142 + 6.4298 * x; r = 0.9229; p < 0.0001; r^2^ = 0.8517) and stipes (y = 0.9553 + 2.7493 * x; r = 0.8907; p < 0.0001; r^2^ = 0.7933) of the fruiting bodies of *B*. *edulis* from the Yunnan Province and soil beneath the fruiting bodies.

**Fig 3:**
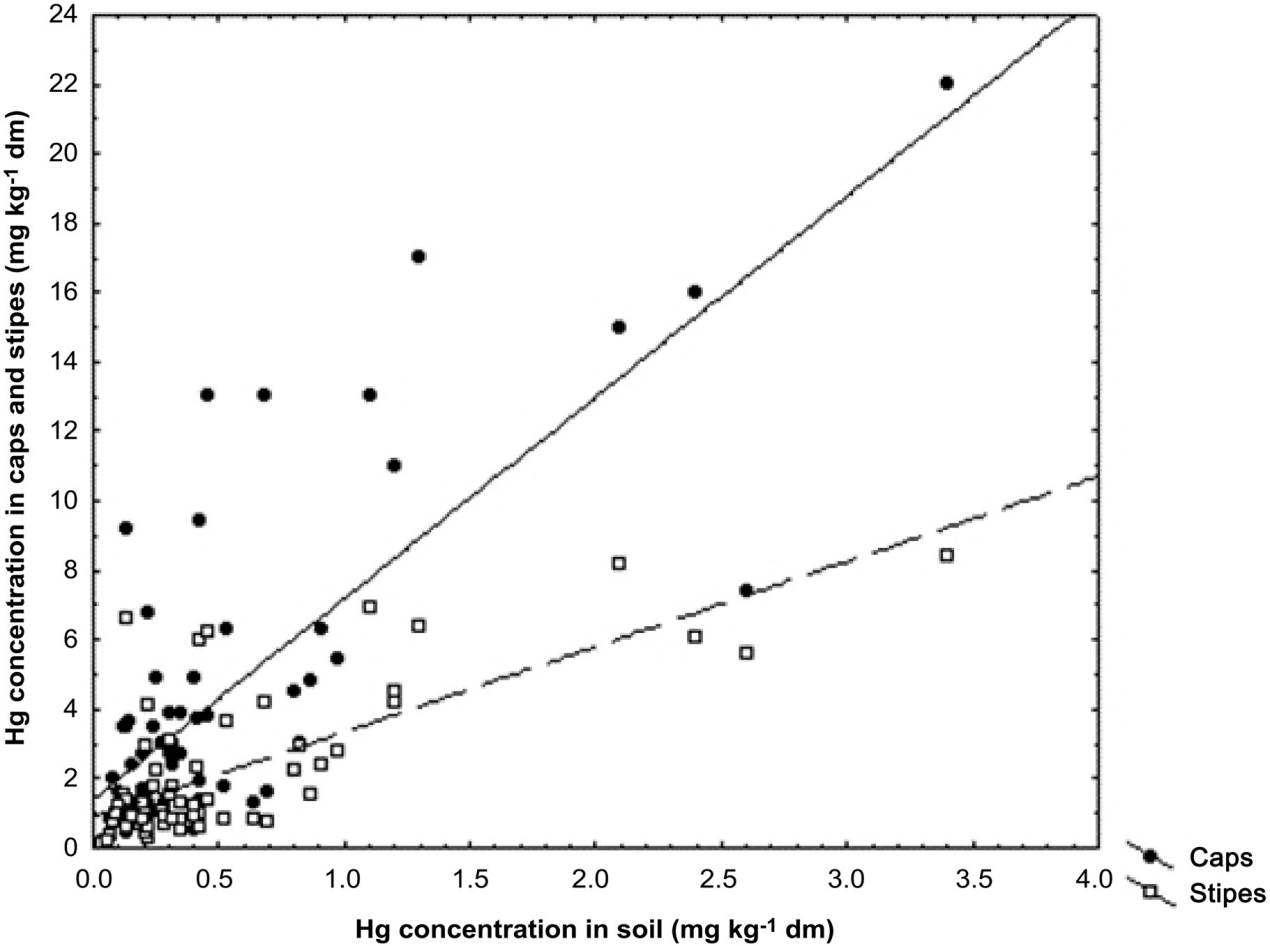
Relationships between Hg concentration in the caps (y = 1.4106 + 5.7865 * x; r = 0.7769 p < 0.0001; r^2^ = 0.6036) and stipes (y = 0.8913 + 2.4557 * x; r = 0.7312; p > 0.0001; r^2^ = 0.5346) of the fruiting bodies of *Boletus* spp. from the Yunnan and Sichuan Provinces and soil beneath the fruiting bodies.

Data obtained in this study imply that *B*. *edulis* is very efficient in mobilization and absorption of sparingly soluble forms of Hg (largely HgS) coming from the geogenic source in forest soil of Yunnan. This is because mushroom *B*. *edulis* has mycelia deeper in soil substratum and Hg concentration accumulated in fruiting bodies by this species seems to be more dependent on the abundance of the geogenic or soil crust Hg from a deeper layer than airborne Hg from a surface layer of soil horizon (this is known from accumulation of ^137^Cs by *B*. *edulis* after the Chernobyl nuclear power plant catastrophe) [84]. The calculated values of Hg bioconcentration factor (BCF), which is a quotient of element in cap, stipe, or a whole fruiting body to soil concentration on dry matter basis, showed that all species for which both the fruiting bodies and samples of soil substratum were available for study showed good potential to accumulate Hg (BCF well above 1). Surprisingly, the values of BCF were relatively high even where substratum was relatively rich in Hg due to geochemical anomaly (Table 1), and Hg concentration of the caps and stipes of the fruiting bodies of *B*. *edulis* positively correlated with concentration in soil—correlation coefficient (*r*) respectively at 0.89 and 0.92 (p < 0.005) (Fig 2). Also a highly significant correlation was determined for relationship between Hg concentration of the caps (0.78; p < 0.0001) and stipes (0.89; p < 0.0001) of the fruiting bodies and soil substratum for all *Boletus* spp. (Fig 3). These results showed that *B*. *edulis* is a potential bioindicator of Hg contained in soil substratum beneath the fruiting bodies and probably especially when the mineral soil horizon is enriched in this element. In a wide study on occurrence and accumulation of Hg and other metallic elements and minerals by *B*. *edulis* across Poland a weak positive relationship could be found only for Cd in fruiting bodies and topsoil but not for Hg [26,27,74,75].

The results from present study and a sufficient number of published data from Europe imply that *B*. *edulis* is able to efficiently up-take (BCF > 1) and sequester Hg in fruiting bodies at elevated concentration when they emerge in the “background” areas (assuming lack of hot spot anthropogenic Hg pollution), while this will be higher if they emerge from the latsols and red and yellow soils which are naturally rich in Hg because of a geochemical anomaly. In other words, the geogenic Hg in mineral layer of soil horizon seems to be for *B*. *edulis* an overriding source of the element than the airborne Hg retained in organic layer above the mineral horizon.

### The probable daily intake (PDI) of Hg with *Boletus* spp. in Yunnan

The Hg concentration in the caps and stipes of *B*. *edulis* ranged respectively from 0.16 to 2.2 mg kg^-1^ fresh product (fp) and 0.085 to 0.84 mg kg^-1^ fp (assuming moisture concentration of 90%), while for a whole set of mushrooms of genus *Boletus* in this study data were more heterogeneous, *i*.*e*. from 0.013 to 2.2 mg kg^-1^ fp in caps and from 0.022 to 0.84 mg kg^-1^ fp in stipes. Based on these figures, popularity of *Boletus* mushrooms in Yunnan and assessed consumption rate by adult (60 kg body mass) as 100 g of fresh caps *per* meal taken up to three times in a week in the mushrooming season and no Hg intake from other sources, the probable dietary intake of Hg is estimated at between 0.016 and 0.22 mg (0.00027 and 0.0037 mg kg^-1^ bm) with one meal composed of 100 g of caps of *B*. *edulis* and at between 0.048 and 0.66 mg (0.00081 and 0.011 mg kg^-1^ bm) with three meals in a week.

The body mass proportion between cap and stipe of an individual fruiting body of *B*. *edulis* changes when it becomes matured with cap getting bigger in mature specimens. People foraging for *Boletus* mushrooms usually collect both young and small in size and mature fruiting bodies. In an attempt aiming to assess probable dietary intake of Hg by individuals eating *Boletus* mushrooms a proportion between body mass of cap to stipe as 1:1 was used.

The value of provisional tolerable weekly intake (PTWI) of Hg is 0.004 mg kg^-1^ bm [85]. The Hg intakes calculated for most of *Boletus* mushrooms in Yunnan were below the PTWI and without health risk, while in a few samples were higher than the standard even if no intake of Hg from other food is considered. Limited is knowledge on impact of cooking on behaviour of Hg contained in fruiting bodies of mushrooms [61,86], and unknown is bio-availability from a meal and bio-accessibility at a cellular level of Hg from edible mushrooms. This is because of a limited knowledge on concentrations of ligands antagonistic to Hg, e.g. Se, S, contained in fruiting bodies of mushrooms and on the molecular forms of Hg and Se and possible effect by Se. Selenium is usually present in elevated concentration in the “real boletes”, *i*.*e*. *Boletus* spp. [56,59], and which in fact efficiently bio-concentrate also Hg. Selenium can have a role as an agent diminishing biological impacts by Hg [16] but is so far little studied or known in case of mushrooms relatively rich in Hg.

## Conclusions

Hg in 21 species of the fruiting bodies of *Boletus* from Yunnan was measured and Hg levels for *B*. *edulis* were found to be elevated over the background levels. The majority of the species have no previous data on Hg content in fruiting bodies from either China or elsewhere. There was a highly significant correlation between Hg content of the caps (0.89; p <0.005) and stipes (0.92; p< 0.005) of the fruiting bodies and Hg content of the soil substratum for *Boletus edulis*. Hg content in the soils is elevated above National Background due to Yunnan being located in the Circum-Pacific Global Mercuriferous Belt.
